# Systematic review and meta-analysis of the efficacy of gabapentin in chronic female pelvic pain without another diagnosis

**DOI:** 10.1016/j.xagr.2021.100042

**Published:** 2021-12-10

**Authors:** Greg Marchand, Ahmed Taher Masoud, Malini Govindan, Kelly Ware, Alexa King, Stacy Ruther, Giovanna Brazil, Kaitlynne Cieminski, Nicolas Calteux, Catherine Coriell, Hollie Ulibarri, Julia Parise, Amanda Arroyo, Diana Chen, Maria Pierson, Rasa Rafie, Katelyn Sainz

**Affiliations:** 1 From the Marchand Institute for Minimally Invasive Surgery, Mesa, AZ (Drs Marchand, Masoud, and Govindan, MsesWare, King, Ruther, Brazil, andCieminski, MrCalteux, MsesCoriell, Ulibarri, Parise, and Arroyo, and Dr Sainz); 2 International University of the Health Sciences, Basseterre, Saint Kitts and Nevis (Ms Ware); 3 Fayoum University Faculty of Medicine, Fayoum, Egypt (Dr Masoud); 4 Chicago College of Osteopathic Medicine, Midwestern University, Glendale, AZ (Mses Chen and Pierson); 5 Rocky Vista University College of Osteopathic Medicine, Parker, CO (MsRafie)

**Keywords:** chronic pelvic pain, gabapentin, meta-analysis, neurontin, pelvic pain

## Abstract

**Background:**

While widely used for the treatment of chronic pelvic pain, limited data exists on efficacy of gabapentin, especially in the subgroup of women suffering from chronic pelvic pain without a known diagnosis, such as endometriosis.

**Objective:**

This study aimed to assess the efficacy of gabapentin when administered to women with chronic pelvic pain without another diagnosis.

**Study Design:**

We performed a Systematic Review and Meta Analysis including all controlled clinical trials addressing the use of gabapentin for the treatment of chronic pelvic pain without another diagnosis. We searched PubMed, Scopus, Web of Science, ClinicalTrials.Gov, MEDLINE, and The Cochrane Library from inception of each database to April 30, 2021. We included all the studies that fulfilled the following criteria: (1) population: women suffering from chronic pelvic pain without another identified diagnosis (such as endometriosis); (2) intervention: gabapentin (regardless of the dosage); (3) comparator:placebo; (4) outcomes: pain score (visual analog scale) after 3 months and pain score (visual analog scale) after 6 months as primary outcomes; and (5) study design: we only included randomized or controlled clinical trials. Our exclusion criteria included (1) uncontrolled clinical trials, (2) studies that did not report data or measures for any of our selected outcomes, (3) studies that included patients with surgically or clinically diagnosed endometriosis, or (4) studies with no full-text manuscript available. Risk of bias assessment was performed using the Cochrane risk of bias tool. We analyzed dichotomous outcomes as percentages and totals, whereas continuous outcomes were analyzed using mean difference, standard deviations, and relative 95% confidence intervals using the inverse variance method.

**Results:**

We included 4 placebo-controlled randomized controlled trials. Analysis was hindered because half of the studies (n=2) used the visual analog scale pain score and the other half (n=2) used the numerical rating scale. The analysis showed that when compared with the placebo, gabapentin significantly lowered the visual analog scale pain score at 3 months (mean difference, 0.79; 1.23 to 0.35; *P*=.005) and 6 months (mean difference, 1.68; 2.30 to 1.05; *P*=.001) and the numerical rating scale pain score at 3 months (mean difference, 0.20; 0.25 to 0.15; *P*=.001). However, in terms of the numerical rating scale pain score after 6 months, the 2 groups showed no significant difference (mean difference, 0.27; 0.80 to 0.26; *P*=.32).

**CONCLUSION:**

Gabapentin may hold benefit for the management of chronic pelvic pain, with significant improvement in pain seen in both scales at 3 months when compared with the placebo, but only in the visual analog scale group at 6 months of usage. Secondary to the differences in the nature of the 2 scales, a further weighted combined analysis was not possible.

**AJOG Global Reports at a Glance**

**Why was this study conducted?**

The authors identified several recent randomized clinical trials that investigated the usage of gabapentin for the treatment of pelvic pain that was not caused by another identified diagnosis, such as endometriosis. Thus, they decided that performing a meta-analysis of these studies might give more information about the use of gabapentin for this purpose.

**Key findings**

The meta-analysis showed gabapentin to be more effective in treating chronic pelvic pain than the placebo at 3 months of usage, however, differences in the 2 different pain scales used by the included studies made it impossible to collectively interpret the data for the 6 month mark.

**What does this add to what is known?**

This study gives some evidence for the use of gabapentin, at least in the short term, for the treatment of pelvic pain without another diagnosis.

## Introduction

Chronic pelvic pain (CPP) can refer to a symptom or diagnosis and affects up to 24% of females worldwide.^1^ It represents a constant or intermittent pain (noncyclic) in the pelvis or the lower abdomen (below the umbilicus) for at least 6 months and is associated with functional disability or requires regular medical care.^1^, ^2^, ^3^ Many factors that may predispose patients to CPP have been described. These include pelvic inflammatory diseases, long cycles, heavy menstrual flow, sexual abuse, alcohol abuse, and psychological disorders.^4^

Indisputably, in CPP patients, a workup is indicated to look for a correctable cause for that patient's pain. This may include an ultrasound, other blood work or imaging studies, and, in many cases, laparoscopy (the gold standard for diagnosing endometriosis) may be indicated.^5^ Estimates of how often an exact diagnosis can be found to explain a patient's pain vary from 45% to 65% with the most common diagnoses being endometriosis, ovarian cysts, pelvic inflammatory disease, and adenomyosis.^5^ Other, nongynecologic causes can include irritable bowel syndrome, painful bladder syndrome, and musculoskeletal disorders.^6^ As a consequence, endometriosis is considered the most common cause of CPP, however, for the large number of patients who continue to have pelvic pain but who have no detected diagnosis, treatment options can be difficult.^6^ Many gynecologists have recommended multispecialty pelvic pain programs to identify other possible causes of pelvic pain, including gastroenterologic causes such is ulcerative colitis or irritable bowel syndrome, urologic conditions such as kidney stones, and rheumatologic conditions such as fibromyalgia.^7^ Even with a complete investigation of these underlying causes by the appropriate medical specialties, in some cases, pain will still persist with no identifiable diagnosis. For these patients, there are only a few options, including continuing to live in pain, opioid pain management, and some alternative therapies such as transcutaneous electrical nerve stimulation, physiotherapy, and naturopathic treatments. Consequently, an additional option, such as a nonopioid medication capable of modulating the central nervous system (CNS) to relieve this pain, would certainly be of benefit.^8^,^9^

Gabapentin (a gamma-aminobutyric acid analog) is an important medication used in the treatment of epilepsy. However, it has been used extensively for pain management in many different chronic pain conditions, including chronic, acute, and postoperative pain,^10^,^11^ diabetic neuropathic pain, postherpetic neuralgia, spinal cord injury, and many other neurologic conditions. Because of its proven efficacy in the treatment of other painful conditions, it is increasingly being used as a treatment for females with CPP.^11^ Neuroimaging studies have shown the effects of gabapentin on brain activity in patients with chronic pain.^12^ The medication can easily pass through the blood-brain barrier where it may then act by inhibiting voltage-gated calcium channels.^13^ Its mechanism of action is not completely understood, but it is thought to modulate the pain pathways in the CNS and to work to stop the phenomenon of central sensitization.^14^

Central sensitization is an enhancement in the function of nerve cells in nociceptive pathways, which is caused by increases in membrane excitability and synaptic efficacy.^15^^,^^16^ It can occur in response to acute pain, inflammation, or neural injury.^17^ The result of this phenomenon is that previously subthreshold synaptic inputs are recruited to nociceptive neuron resulting an increased action potential. Therefore, within the CNS, acute pain from any source can become chronic in nature. Because of its proposed mechanism of action, gabapentin may be particularly useful in the treatment of patients suffering from this phenomenon.^17^

Therefore, in this systematic review and meta-analysis, we aimed to include the highest quality data available to date to assess the efficacy of gabapentin in patients suffering from CPP without any other diagnosis. The authors are not aware of or could not find any previous systematic reviews or meta-analyses that specifically focused on this outcome.

## Materials and Methods

This meta-analysis was performed according to the Preferred Reporting Items for Systematic Reviews and Meta-Analyses (PRISMA)^18^ and the guidelines reported in the Cochrane Handbook for Systematic Reviews of Interventions.^19^ The study was registered with the International Prospective Register of Systematic Reviews (identification number, CRD42021247474).

### Literature search

We searched 6 databases, namely Web of Science, Scopus, Cochrane Central Register of Controlled Trials, ClinicalTrials.Gov, MEDLINE, and PubMed from inception through March 31, 2021. We followed the following search strategy with no restriction on time or languages: (“Gabapentin” OR “Gabarone” OR “Gralise” OR “Neurontin” OR “Fanatrex”) AND (“chronic pelvic pain” OR “CPP” OR “endometriosis”).

### Eligibility criteria

We included all the studies that fulfilled the following criteria: (1) population: women suffering from CPP (defined as pain for >6 months,) without another identified diagnosis, (such as endometriosis); (2) intervention: gabapentin (regardless of the dosage); (3) comparator: placebo; (4) outcomes: pain score (visual analog scale [VAS]) after 3 months and pain score (VAS) after 6 months as primary outcomes. The secondary outcomes were pain score (numerical rating scale [NRS]) after 3 months and pain score (NRS) after 6 months. (5) Study design: we included only clinical trials. Our exclusion criteria were (1) uncontrolled clinical trials, (2) studies that did not report data or measures for our selected outcomes, (3) studies that did not exclude patients with diagnosed endometriosis, or (4) studies for which no full-text manuscript was available.

### Screening of results

We exported the results of the search using Endnote X8.0.1 (Build 1044) (Clarivate Analytics, London, United Kingdom), which included 193 records following the removal of 28 duplicate records. Thereafter, we screened the studies manually in 2 steps, namely a title and abstract screening followed by a full-text screening. Two authors performed the screening, with a third author to assist if any disagreement presented. Ultimately, 4 studies met our inclusion criteria and were included in our quantitative synthesis.

### Data extraction and analysis

After the screening step, we extracted the data from the selected studies and categorized the data into the following 3 main groups: (1) baseline and demographic data of patients in each study, including age (years), body mass index (BMI) (kg/m^2^), parity, duration of CPP (months), and previous pelvic surgery; (2) data for analysis including outcome values of VAS after 3 months, VAS scores after 6 months, NRS scores after 3 months, and NRS scores after 6 months. In addition to the previous 2 categories, we extracted data for the 7 domains assessing the risk of bias (ROB) according to Cochrane's ROB tool.^23^

### Data analysis

We used the Cochrane Review Manager Software (RevMan 5.4.1) to perform our analysis. We analyzed dichotomous outcomes using percentage and total, whereas continuous outcomes were analyzed using the mean difference (MD), standard deviations (SDs), and relative 95% confidence interval (CI) using the inverse variance method. To test for heterogeneity among studies, the *I*^2^ statistic and the *P* value of the chi-square test were used. Outcomes with *I*^2^ >50% and *P*<.1 were considered heterogeneous, whereas outcomes with *I*^2^ <50% and *P*>.1 were considered homogeneous according to the Cochrane Handbook. Homogenous data were analyzed using a fixed effects model, whereas heterogeneous outcomes were analyzed using the random effects model.

### Quality assessment

We evaluated the quality of this systematic review and meta-analysis using the Grading of Recommendations Assessment, Development and Evaluation (GRADE) guidelines. We included only randomized controlled trials (RCTs) and excluded uncontrolled and observational studies. We then evaluated the quality of this systematic review and meta-analysis using the Grading of Recommendations Assessment evaluation tool. In accordance with the Cochrane ROB tool for clinical trials, we performed a ROB investigation for the included studies. The domains assessed included (1) proper randomization, (2) blinding to the allocation of patients into each group, (3) type of blinding (single, double or none), (4) attrition bias, (5) selection bias, (6) blinding of the assessor of outcomes, and (7) other bias. We also assessed the total ROB for all of the studies. Two authors collaborated to grade each domain for each study as high, low, or unclear ROB. In any case of disagreement, a third author was consulted to achieve consensus.

## Results

### Summary of included studies

**Figure 1:**
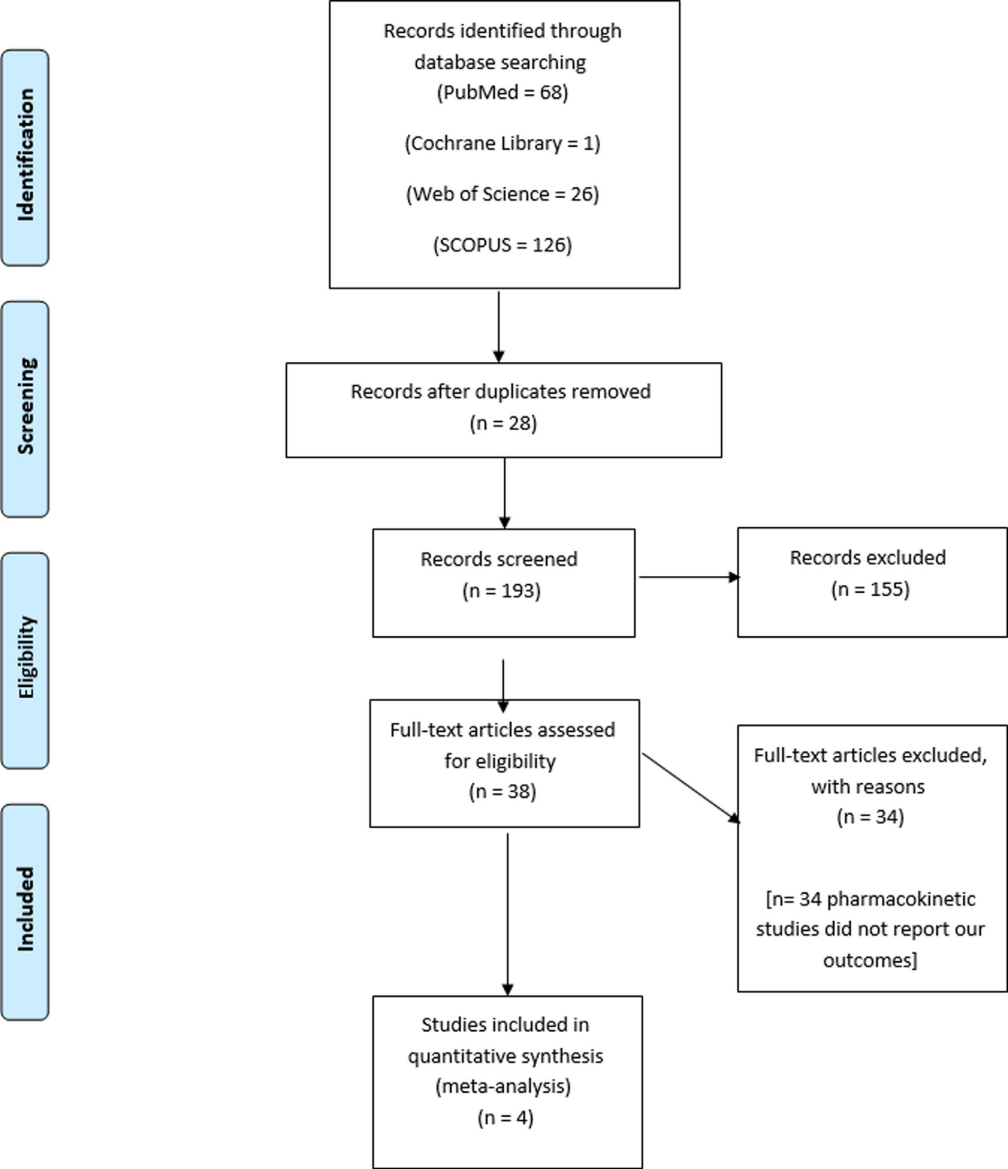
PRISMA flow diagram of our literature search *PRISMA*, Preferred Reporting Items for Systematic Reviews and Meta-analysis.

Figure 1 shows a PRISMA flow diagram of our literature search. In our study, we performed an analysis of 425 patients from 4 studies.^21^, ^22^, ^23^, ^24^ A total of 211 patients were allocated to a gabapentin group for CPP and 214 patients were allocated to a placebo group. The mean age of the gabapentin group was 30.5±7.7 years, whereas that of the placebo group was 30.1±8.6 years. The Table shows a detailed summary of the included participants and their demographic data, including age (years), BMI (kg/m^2^), parity, duration of CPP (months), and previous pelvic surgery.

Marchand. Meta-analysis of gabapentin in chronic pelvic pain. Am J Obstet Gynecol Glob Rep 2022.

### Results of risk of bias assessment

**Figure 2:**
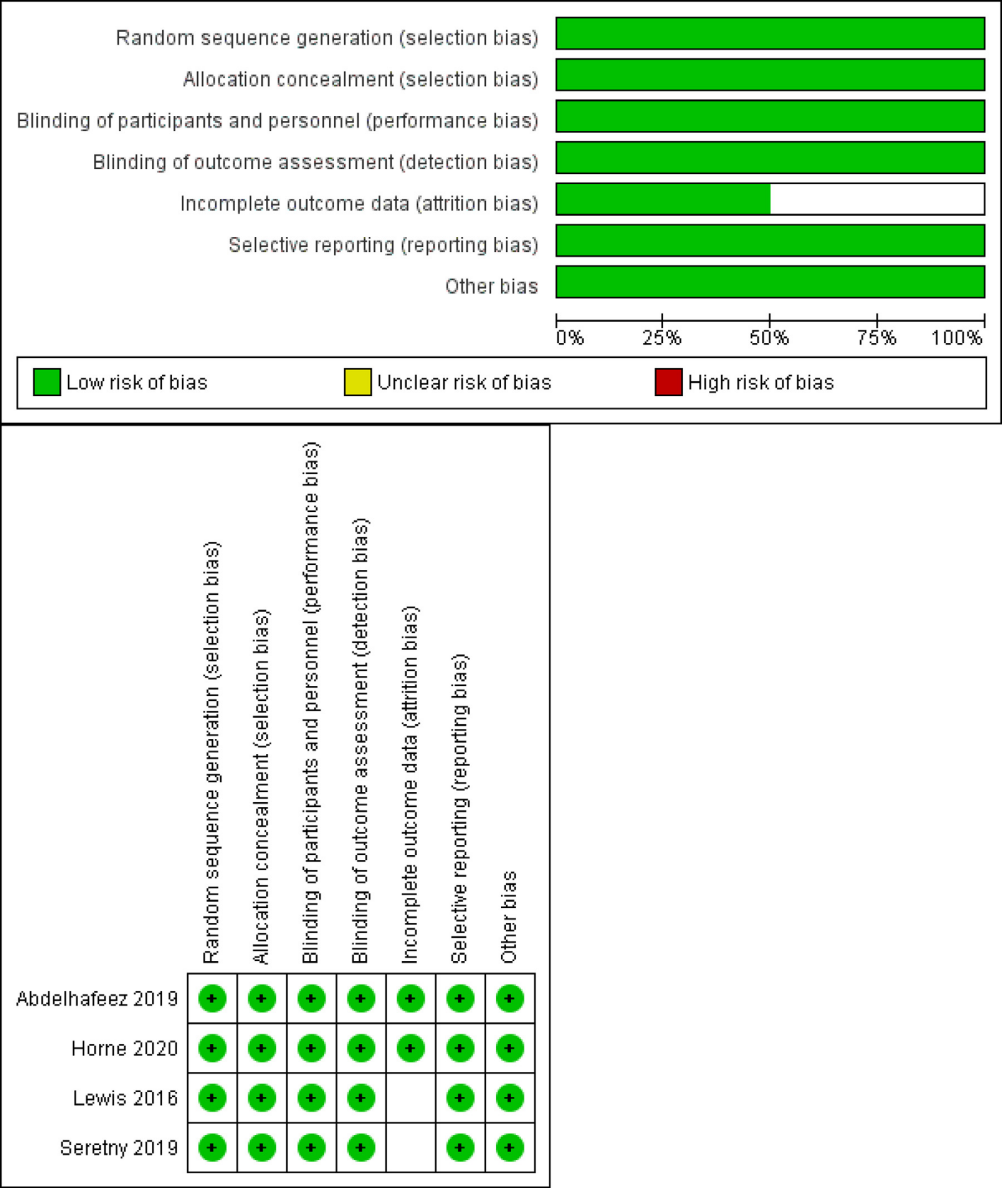
Risk of bias assessment

The result of the ROB assessments yielded an overall low ROB according to the Cochrane tool. Regarding randomization, all studies were at low ROB. As for the allocation concealment, all studies reported adequate allocation concealment; therefore, they were judged to have a low ROB. All studies were judged to have a low ROB in the category of blinding of the participants and personnel, and all studies were judged to be at low ROB in the blinding of the outcome assessment. For the remaining domains of the Cochrane tool, an allocation of low ROB were given, except for 2 studies^22^^,^^24^ that failed to report enough information about their risk of selection bias and therefore they were judged to have unclear ROB. A summarized illustration of the ROB of included trials is seen in Figure 2, and the complete details of the risk of bias assessment can be found in the Supplemental Table.

Marchand. Meta-analysis of gabapentin in chronic pelvic pain. Am J Obstet Gynecol Glob Rep 2022.

### Pain score (visual analog scale) after 3 months

**Figure 3:**
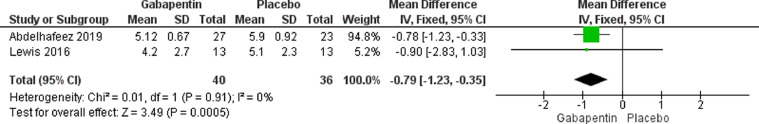
Forest plot for the analysis of the VAS pain score after 3 months *CI*, confidence interval; *IV*, inverse variance; *SD*, standard deviation; *VAS,* visual analog scale.

Pain scores (VAS) after 3 months were reported in 2 studies.^21^^,^^24^ There was a significant difference in the overall MD between the 2 groups (MD, −0.79; −1.23 to −0.35; *P*=.005). The pooled analysis was homogeneous (*P*=.91; *I*^2^=0%) as can be seen in Figure 3.

Marchand. Meta-analysis of gabapentin in chronic pelvic pain. Am J Obstet Gynecol Glob Rep 2022.

### Pain score (visual analog scale) after 6 months

**Figure 4:**
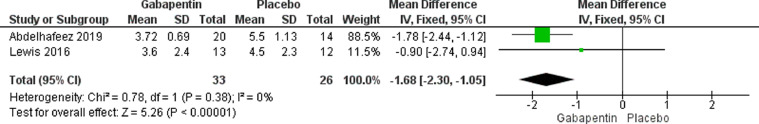
Forest plot for the analysis of the VAS pain scores after 6 months *CI*, confidence interval; *IV*, inverse variance; *SD*, standard deviation; *VAS,* visual analog scale.

Two studies^21^^,^^24^ reported pain scores (VAS) after 6 months. There was a significant difference in the overall MD between the 2 groups (MD, −1.68; −2.30 to −1.05; *P*=.001). The pooled analysis was homogeneous (*P*=.38; *I*^2^=0%) as can be seen in Figure 4.

Marchand. Meta-analysis of gabapentin in chronic pelvic pain. Am J Obstet Gynecol Glob Rep 2022.

### Pain score (numerical rating scale) after 3 months

**Figure 5:**
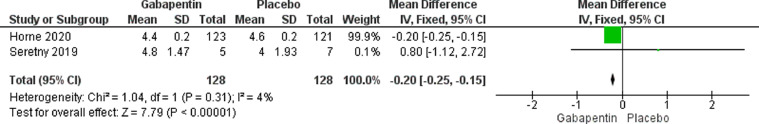
Forest plot for the analysis of the NRS pain scores after 3 months *CI*, confidence interval; *IV*, inverse variance; *NRS*, numerical rating scale; *SD*, standard deviation.

Two studies^22^^,^^23^ reported pain scores (NRS) after 3 months. The overall MD showed that there was a significant difference between the 2 groups (MD, −0.20; −0.25 to −0.15; *P*=.001). The pooled analysis was homogeneous (*P*=.31; *I*^2^=4%) as can be seen in Figure 5.

Marchand. Meta-analysis of gabapentin in chronic pelvic pain. Am J Obstet Gynecol Glob Rep 2022.

### Pain score (numerical scale rating) after 6 months

**Figure 6:**
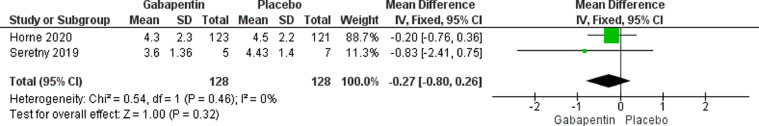
Forest plot for the analysis of the NRS pain scores after 6 months *CI*, confidence interval; *IV*, inverse variance; *NRS*, numerical rating scale; *SD*, standard deviation.

Two studies^23^^,^^24^ reported the pain scores (NRS) after 6 months. The overall MD showed that there was no significant difference between the 2 groups (MD, −0.27; −0.80 to 0.26; *P*=.32). The pooled analysis was homogeneous (*P*=.46; *I*²=0%) as can be seen in Figure 6.

Marchand. Meta-analysis of gabapentin in chronic pelvic pain. Am J Obstet Gynecol Glob Rep 2022.

## Discussion

We included 425 patients from 4 clinical trials. All trials excluded any patients with a previous diagnosis explaining their pain. We found that gabapentin significantly reduced the pain score after 3 and 6 months when measured using the VAS scale compared with the placebo group. In addition, it also reduced the pain score after 3 months using the NRS scale in comparison with the placebo. However, there was no significant difference for either of the groups in terms of pain score after 6 months using the NRS scale. Unfortunately, because of the differences inherent to these 2 pain scales (NRS and VAS), a perfect direct comparison cannot be made to pool the outcomes, and, consequently, our results are somewhat conflicting.

Table 1

**Table 1:** Detailed summary of the included patients, their demographic data, body mass index, previous pelvic surgery, parity, and duration of chronic pelvic pain

	Sample size (n)		Age (y), mean (SD)		Duration of chronic pelvic pain (mo), mean (SD)		BMI (kg/m^2^), mean (SD)		Parity, mean (SD)		Previous pelvic surgery, n (%)	
Study ID	Gabapentin	Placebo	Gabapentin	Placebo	Gabapentin	Placebo	Gabapentin	Placebo	Gabapentin	Placebo	Gabapentin	Placebo
Lewis et al,^24^ 2016	22	25	nr	nr	nr	nr	nr	nr	nr	nr	nr	nr
Abdelhafeez et al,^21^ 2019	30	30	32.7 (4.91)	30.27 (5.32)	15.7 (7.4)	18 (5.9)	28.37 (4.67)	28.35 (4.88)	3 (1.5)	2.7 (2.2)	5 (16.7)	2 (6.67)
Seretny et al,^22^ 2019	6	6	0 (14.2)	0 (9)	nr	nr	0 (5.06)	0 (6.06)	nr	nr	nr	nr
Horne et al,^23^ 2020	153	153	30.5 (7.7)	30.1 (8.6)	nr	nr	27.1 (5.7)	27.8 (5.9)	nr	nr	nr	nr

*BMI*, body mass index; *nr*, not reported; *SD*, standard deviation.

Before conducting their multicentric, placebo-controlled RCT on gabapentin, Horne et al^23^ observed the increased prescription of gabapentin for CPP in his surrounding community, with the rate of prescription tripling in the time period from 2007 to 2017.^20^ They proceeded to survey 2 random groups of general practitioners and gynecologists and found that 74% of the general practitioners and 92% of the gynecologists would prescribe gabapentin as a treatment option for CPP. This indicates how widely the medication is used, even without robust evidence for its efficacy, which, of course, provided the impetus to conduct RCTs such as the aforementioned studies and was one of the main inspirations for us to perform this analysis.

Regarding the adverse effects of the gabapentin, AbdelHafeez et al^21^ reported that dizziness was the only major side effect that was seen more frequently in the gabapentin group and commented that other adverse effects did not differ significantly between the intervention and control groups. However, not all trials on gabapentin had the same finding. Drowsiness and visual disturbances also were found to be significantly increased in the gabapentin groups according to the findings of Horne et at^23^ and Moore at al.^25^ A review of the side effects is beyond the scope of this study and something the authors would like to consider in the future; however, the fact that all 4 included studies had a very low side-effect rate for the administered gabapentin may suggest that it is also well tolerated in the CPP population.

Although the demonstrated efficacy of gabapentin in our meta-analysis was encouraging, the confounding findings still do not give us a clear answer on the longer-term benefit of this medication for CPP management. More trials will be needed to fully understand the long-term benefit or lack thereof.

### Strengths

The strengths of our meta-analysis included the fact that we conducted this study in adherence with the Cochrane Handbook.^19^ Next, all the included studies were homogenous and we included only placebo-controlled RCTs, which seemed to be well designed and sufficiently powered for their stated goal. This ensures the strongest evidence according to the GRADE. In addition, we tried to cover 2 follow-up periods, which gives more reliable evidence regarding clinical outcomes. Finally, most studies showed a low ROB in nearly all the assessed domains.

### Limitations

The sample size in each trial represents the major limitation, with only 4 studies and 425 patients included. Because our sample size was relatively small, most of our participants came from only 1 trial (Horne et al^23^). The unfortunate result, combined with pain scales that could not be perfectly combined, was insufficient data to prove a difference for 1 of our outcomes. Another limitation was the duration of the trials included, which was short relative to the very diagnosis of CPP that requires 6 months of pelvic pain. Another limitation included the lack of information on the duration of CPP beyond 6 months and the lack of data necessary to further categorize results based on the dosage of gabapentin. Therefore, we would recommend that future RCTs should consider the possibility of longer-term follow-ups. We recommend further research on the medical management of CPP, especially on combination therapy of gabapentin with other agents instead of gabapentin monotherapy and the introduction of multiple dosing regiments.

## Conclusion

Gabapentin may be an effective agent in the management of CPP, at least for the treatment of CPP in the first 3 months. Confounding findings involving different RCTs using different pain scales will limit a true understanding of the effect on pain at 6 months of treatment until further RCTs are performed.

## Appendix Supplementary materials

Image, application 1
