# Supplementary material for: Systematic review and meta-analysis of the efficacy of gabapentin in chronic female pelvic pain without another diagnosis
Source: AJOG Glob Rep. 2021 Dec 10;2(1):100042. doi: 10.1016/j.xagr.2021.100042 (PMC9563541; doi:10.1016/j.xagr.2021.100042)
Supplement: Supplementary file 1 [file mmc1.docx]

**Supplementary Table 1:** Details of the risk of bias assessment.

| study | Randomization | Allocation  Concealment | Blinding of  participants | Blinding of  assessors of  outcomes | Attrition  bias | Selection  bias | Other  bias |
| --- | --- | --- | --- | --- | --- | --- | --- |
| Lewis 2016 | low | low | low | low | unclear | low | low |
| Abdelhafeez 2019 | low | low | low | low | low | low | low |
| Seretny 2019 | low | low | low | low | unclear | low | low |
| Horne 2020 | low | low | low | low | low | low | low |
